# Diet Induced Obesity Alters Intestinal Cytoplasmic Lipid Droplet Morphology and Proteome in the Postprandial Response to Dietary Fat

**DOI:** 10.3389/fphys.2019.00180

**Published:** 2019-03-05

**Authors:** Theresa D’Aquila, Alyssa S. Zembroski, Kimberly K. Buhman

**Affiliations:** Department of Nutrition Science, Purdue University, West Lafayette, IN, United States

**Keywords:** obesity, cytoplasmic lipid droplet, dietary fat absorption, triacylglycerol, lipid droplet proteome, enterocytes

## Abstract

Dietary fat absorption by the small intestine is an efficient, multistep process that regulates the uptake and delivery of essential nutrients and energy. Fatty acids taken up by enterocytes, the absorptive cells of the small intestine, are resynthesized into triacylglycerol (TAG) and either secreted in chylomicrons or temporarily stored in cytoplasmic lipid droplets (CLDs). Proteins that associate with CLDs are thought to regulate the dynamics of TAG storage and mobilization. It is currently unclear what effect diet induced obesity (DIO) has on the balance between dietary fat storage and secretion. Specifically, there is limited knowledge of how DIO affects the level and diversity of proteins that associate with CLDs and regulate CLD dynamics. In the current study, we characterize CLDs from lean and DIO mice through histological and proteomic analyses. We demonstrate that DIO mice have larger intestinal CLDs compared to lean mice in response to dietary fat. Additionally, we identified 375 proteins in the CLD fraction isolated from enterocytes of lean and DIO mice. We identified a subgroup of lipid related proteins that are either increased or unique to the DIO CLD proteome. These proteins are involved in steroid synthesis, TAG synthesis, and lipolysis. This analysis expands our knowledge of the effect of DIO on the process of dietary fat absorption in the small intestine (D’Aquila, 2016).

## Introduction

Enterocytes, the absorptive cells of the small intestine, are responsible for the uptake, repackaging, and secretion of dietary fat. In addition, enterocytes are capable of temporarily storing dietary fat in CLDs in the postprandial response to dietary fat (reviewed in Beilstein et al., 2016; D’Aquila et al., 2016). Dietary fat in the form of TAG is digested in the lumen of the small intestine producing free fatty acids and monoacylglycerols, which are incorporated into mixed micelles. Mixed micelles interact with the brush border membrane of enterocytes where free fatty acids and monoacylglycerols are absorbed. In the enterocyte, free fatty acids and monoacylglycerols are resynthesized into TAG by acyl-CoA: monoacylglycerol acyltransferase and DGAT at the endoplasmic reticulum (ER). The resulting TAG can be packaged onto a chylomicron particle for secretion or temporarily stored in CLDs. While there are structural and composition similarities between chylomicron particles and CLDs, these lipid containing particles differ in their synthesis and metabolism that allows chylomicrons to be secreted and requires further metabolism of CLDs before the stored lipids are used within or eventually secreted from cells (reviewed in Demignot et al., 2014).

Cytoplasmic lipid droplets are composed of a neutral lipid core, a phospholipid monolayer, and associated proteins (Beilstein et al., 2016). Proteins that associate with CLDs are thought to regulate the storage and mobilization of TAG. Proteins associated with CLDs isolated from enterocytes after a lipid challenge have been identified by targeted (Seyer et al., 2013) and global proteomic (Bouchoux et al., 2011; Beilstein et al., 2013; D’Aquila et al., 2015) approaches. These studies have identified and confirmed that members of the Perilipin (Plin) family (Plin2 and Plin3) associate with CLDs. Interestingly, these studies have also identified proteins associated with a wide variety of biological pathways including lipid catabolic and anabolic pathways.

The effect of obesity on intestinal lipid metabolism is currently unclear; however, research suggests that it is dysregulated in this metabolic state as well as in other disease states (reviewed in Dash et al., 2015; Xiao et al., 2018). Recent studies in high fat diet fed (non-obese), *ob/ob* mice, and DIO mice have demonstrated decreased TAG secretion rates in the postprandial response to dietary fat (Douglass et al., 2012; Uchida et al., 2012). In addition, DIO alters intestinal mRNA levels of proteins involved in chylomicron assembly, TAG synthesis, and fatty acid trafficking (Uchida et al., 2012).

Little is known of the effect of obesity on CLD morphology and associated proteins in enterocytes. DIO alters the CLD proteome in adipocytes and hepatocytes (Ding et al., 2012; Khan et al., 2015). Previous studies have shown alterations in mRNA levels of proteins known to associate with CLDs including lipases (Uchida et al., 2012) and members of the Plin family (Lee et al., 2009). In enterocytes, Plin2 and 3 localize to CLDs in the postprandial response to dietary fat; however, their localization varies under different physiological conditions. Plin3 localizes to CLDs in response to a dietary fat challenge defined as 2 h after administration of a 200 μl olive oil oral gavage. Plin2 localizes to CLDs in response to chronic high fat feeding in a DIO mouse model (Lee et al., 2009). A comprehensive analysis of proteins associated with CLDs from lean and DIO mice in the postprandial response to dietary fat has not been conducted.

To determine the effects of DIO on enterocyte CLD morphology and proteome in the postprandial response to dietary fat, we investigated CLDs within enterocytes of lean and DIO mice given an oil bolus orally by transmission electron microscopy, immunofluorescence microscopy, and proteomic analysis. Based on previous observations of altered TAG secretion in DIO mice in response to a dietary fat challenge (Douglass et al., 2012; Uchida et al., 2012), we hypothesize that DIO mice have altered CLD morphology and CLD proteome.

## Materials and Methods

### Mice

All animal experiments were carried out in accordance with the National Institute of Health Guide for the Care and Use of Laboratory Animals and approved by the Purdue Animal Care and Use Committee. C57BL/6 male mice from an in-house breeding colony were used for this study. The mice were fed a chow diet (PicoLab 5053, Lab Diets, Richmond, IN, United States) that consists of 62.1% of calories from carbohydrate (starch), 24.7% from protein, and 13.2% from fat from weaning to 5 weeks of age. The mice were housed in a temperature and humidity controlled facility with a 12 h light/dark cycle (6AM/6PM) with *ad libitum* access to food and water.

### Generation of DIO Mouse Model

C57BL/6 male mice were fed either high fat or low fat diets. The high fat diet (D12492, Research Diet, New Brunswick, NJ, United States) consists of 20% of calories from carbohydrates, 20% from protein, and 60% from fat. The low fat, D12492 matched diet (D12450J, Research Diet, New Brunswick, NJ, United States) consists of 70% of calories from carbohydrates, 20% from protein, and 10% from fat. The mice were fed the diet for 12 weeks and body weights were monitored weekly.

### Mouse Procedure for CLD Isolation and Proteomic Analysis

Five mice from the low fat diet group and five mice from the high fat diet group were fasted for 4 h at the beginning of the light cycle. An oral gavage of 200 μl olive oil was administered and 2 h later, the mice were euthanized via CO_2_ asphyxiation. The small intestine was excised and divided into three equal length segments. The middle segment, representing the jejunum, was used for the analysis.

### Enterocyte Isolation

Enterocytes were isolated from the middle section of the small intestine as previously described (Xie et al., 2003; Lee et al., 2009; D’Aquila et al., 2015). Briefly, the intestinal sections were washed in tissue buffer (Hank’s Balanced Salt Solution with 25 mM HEPES and 1% fetal calf serum) and then placed in isolation buffer (Calcium and magnesium free Hank’s Balanced Salt Solution with 1.5 mM EDTA). The intestine segments were incubated for 15 min at 37°C with rotation. The sample was vortexed briefly and the supernatant containing enterocytes was removed and saved. The process was repeated and the supernatants containing isolated enterocytes were combined.

### CLD Isolation

Cytoplasmic lipid droplets were isolated from enterocytes using a previously established sucrose gradient ultracentrifugation protocol (D’Aquila et al., 2015). Enterocytes were lysed in ice-cold sucrose lysis buffer (175 mM sucrose, 10 mM HEPES, and 1 mM EDTA pH 7.4). Cells were disrupted by passing through a 27-gauge 1-inch needle, eight times. The resulting 2 ml of cell lysate were carefully layered with 6 ml of sucrose-free lysis buffer and centrifuged at 20,000 × *g* at 4°C for 2 h. After centrifugation, the sample was frozen at -80°C. The frozen sample was sliced into seven sequential fractions that were approximately 1 cm in length. The top fraction was used as the isolated CLDs. This procedure was previously validated by immunoblotting for cell fraction specific antibodies and negative stain electron microscopy of isolated CLDs (D’Aquila et al., 2015).

### In-Solution Digestion and LC-MS/MS

In-solution digestion and LC-MS/MS were completed using a previously established protocol (D’Aquila et al., 2015). In preparation for proteomic analysis, the isolated CLD fractions were delipidated using 2:1 chloroform methanol and proteins precipitated using ice-cold acetone. The protein pellet was denatured using 8 M urea and 10 mM DTT for 1.5 h at 37°C. The sample was digested for 12 h using trypsin (Sigma-Aldrich, St. Louis MO, United States) using a ratio of 1 μg trypsin to 50 μg protein which was isolated from the CLD fraction. The reaction was quenched using trifluoroacetic acid. Tryptic peptides were separated on a nanoLC system (1100 Series LC, Agilent Technologies, Santa Clara, CA, United States). The peptides were loaded on the Agilent 300SB-C18 enrichment column for concentration and the enrichment column was switched into the nano-flow path after 5 min. Peptides were separated with a C18 reversed phase ZORBAX 300SB-C18 column. The column was connected to the emission tip and coupled to the nano-electrospray ionization source of the high resolution hybrid ion trap mass spectrometer LTQ-Orbitrap LX (Thermo Fisher Scientific, Waltham MA, United States). The LTQ-Orbitrap mass spectrometer was operated in the data-dependent positive acquisition mode in which each full MS scan (30.000 resolving power) was followed by six MS/MS scans where the six most abundant molecular ions were selected and fragmented by collision induced dissociation (CID) using a normalized collision energy of 35%.

### Protein Identification

The peak list files containing MS and MS/MS data were analyzed using a previously described protocol with MaxQuant version 1.4.08 (Cox and Mann, 2008; Cox et al., 2014; D’Aquila et al., 2015). For protein identification, the MS/MS data was searched against the Uniprot protein database (UniProt Consortium, 2014). The database was searched using the MASCOT search engine utilizing Andromeda as the peptide search algorithm that is incorporated in the MaxQuant platform (Cox et al., 2011). The search was conducted using the following settings: trypsin cleavage with a maximum of two missed cleavages, fixed modification of iodoethanol addition to cysteine, variable modification of oxidation of methionine, and acetylation of the N-terminal. The MS mass tolerance was set at 4.5 ppm with a maximum number of five modifications. The false discovery rate was set at 0.01 for proteins and peptides and was run against a decoy revert database. Peptides required a minimum length of seven amino acids. The MS/MS tolerance was set at 0.1 Da for protein identification. The minimum score for modified and unmodified peptides was set at forty. At least two peptides were required for protein identification. The bioinformatics and statistical package Perseus 1.4.1.3 was used to analyze the MaxQuant output. Contaminants identified by Perseus, such as keratin, were removed from the analysis. For label free quantitation (LFQ), the intensity for each protein was transformed log2(x). To identify the relative level of a specific protein, MaxQuant algorithm compares the signal intensity of a peptide from the protein of interest to the intensity of all peptides in the sample. The resulting level indicates the relative intensity of the protein in the isolated CLD fraction. A protein was considered identified in a diet group if it was identified in at least three out of the five biological replicates. Differences in relative levels of proteins identified in the CLD fraction of enterocytes between lean and DIO mice determined by *t*-test, *p* < 0.05 considered statistically significant.

### Data Analysis and Bioinformatics

The GO terms for biological process associated with identified proteins were determined using GO Enrichment Analysis and Visualization Tool (Gorilla; Eden et al., 2009). Visualization of enriched GO terms was accomplished using GOrilla comparing the isolated CLD proteomic profile to the entire mouse proteome obtained from the Uniprot protein database. The *p*-value threshold was set at *p* < 0.01. Lipid related GO terms were identified and proteins associated with the lipid related GO terms were compiled and are here on referred to as “lipid related proteins.” Visualization of protein interactions was accomplished using STRING version 10.0 (Szklarczyk et al., 2015). We used the confidence view with a score of 0.4, indicating a medium confidence level.

### Immunofluorescence Confocal Microscopy

Immunofluorescence confocal microscopy was completed using a previously described protocol (D’Aquila et al., 2015). Four mice from the low fat diet group and the high fat diet group were fasted for 4 h at the beginning of the light cycle and then administered a 200 μl olive oil bolus. A small (5 mm) section of the jejunum was harvested from the mice 2 h after the dietary fat challenge and was frozen in optimal cutting temperature embedding media in 2-methyl butane cooled with dry ice. The tissue was stored at -80°C until processed for immunofluorescence microscopy. The tissue was sliced into 15 μm tissue sections, fixed in 2% paraformaldehyde, permeabilized with 0.1% saponin, and blocked with 3% bovine serum albumin in PBS. The tissue was probed with previously validated antibodies for Plin3 and Plin2 (Wolins et al., 2005; a gift from Dr. Perry Bickel at the University of Texas Southwestern, Dallas, TX, United States). The sections were also stained for neutral lipids using 1 μg/ml 4,4-difluoro-1,3,5,7,8-pentamethyl-4-bora-3a,4a-diaza-s-indacene (BODIPY; Life technologies, Grand Island, NY, United States), a secondary AlexaFluor antibody (Life Technologies), and for nuclei using 300 nM DAPI (Life Technologies) and imaged using a Nikon A1R confocal microscope (Nikon Instruments Inc., Melville, NY, United States). The enterocytes analyzed were located midway between the crypt and tip of the villi. Image processing was conducted using NIS-Elements C acquisition and analysis software.

### Cardiac Perfusion, Fixation, and Transmission Electron Microscopy

Cardiac perfusion, fixation, and transmission electron microscopy were completed using a previously described protocol (D’Aquila et al., 2015). Three mice from the low fat diet group and high fat diet group were fasted for 4 h at the start of the light cycle. Mice were administered an oral gavage of 200 μl olive oil. Two hours post bolus, the mice were anesthetized using inhaled isoflurane and perfused with 1.5% glutaraldehyde in 0.1 M sodium cacodylate via cardiac infusion. Samples from the jejunum section of the small intestine were isolated, stained with osmium tetroxide, dehydrated, and embedded in resin. Ultrathin sections were stained with lead citrate and uranyl acetate and examined using a Tecnai T20 Transmission Electron Microscope (FEI, Hillsboro, OR, United States).

### CLD Size Analysis

Thirty cells were examined from three mice from each diet group. The cells analyzed were located in the middle section of the villi and at least three villi were measured per mouse. CLD diameter was measured using ImageJ. Image stitching was performed using Autostitch. Data are expressed as the average of the biological replicates ± SEM. Statistical significance was determined using a Type 3 mixed model for fixed effects using SAS v 9.4; *p* < 0.05 was considered significant. CLD size distribution was analyzed by two-sample Kolmogorov–Smirnov test; *p* < 0.05 was considered significant.

## Results

**FIGURE 1:**
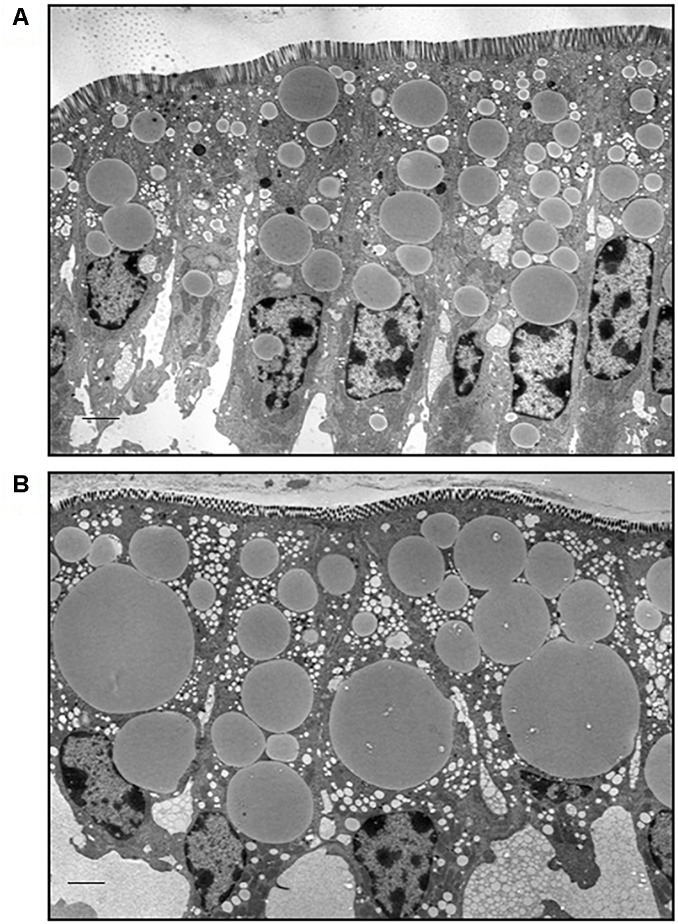
Lipids accumulate in CLDs in enterocytes from lean and DIO mice in the postprandial response to a dietary fat challenge. Representative TEM images from the jejunum section of the intestine from lean **(A)** and DIO **(B)** mice in response to 200 μl olive oil, *n* = 3 mice. Scale bar is 2 μm.

**FIGURE 2:**
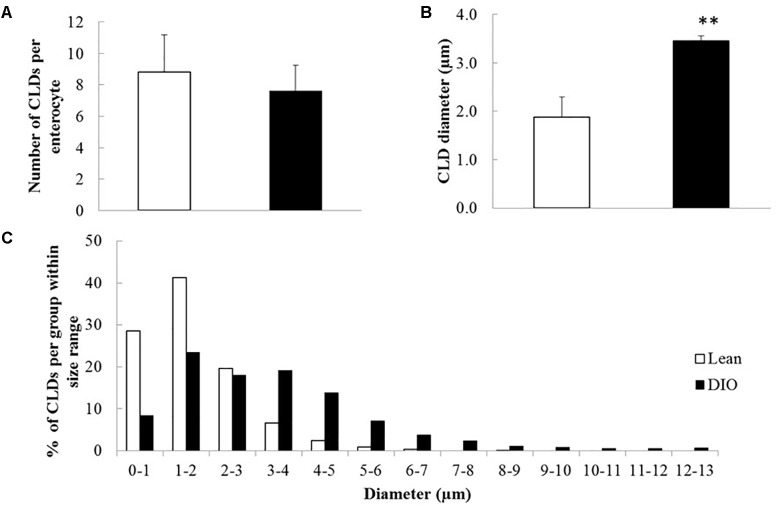
CLDs from enterocytes of DIO mice have a larger average diameter and a larger maximum diameter compared to lean mice in the postprandial response to a dietary fat challenge. CLD diameter and number per cell were determined by analyzing 30 cells per mouse, *n* = 3 mice. **(A)** Average CLD number per enterocyte. No significant difference in number of CLDs per cell between lean and DIO (8.81 CLDs per cell in lean and 7.68 CLDs per cell in DIO mice). **(B)** CLD diameter from lean and DIO mice in the postprandial response to a dietary fat challenge. The average CLD from DIO mice is larger than lean mice in the postprandial response to a dietary fat challenge (^∗∗^*p* < 0.01). The average CLD diameter in DIO mice is 3.35 μm compared to 1.88 μm average CLD diameter in lean mice. Statistical significance was determined by mixed model ANOVA to test for diet effect. **(C)** CLD size distribution from lean and DIO mice. CLD diameter distribution is different between lean and DIO mice (*p* < 0.001). Comparison of CLD size distribution was conducted using a two-sample Kolmogorov–Smirnov test.

Male C57BL/6 mice fed a high fat diet (60% kcal from fat) gained more weight compared to mice fed a low fat diet (10% kcal from fat) for 12 weeks (Supplementary Figure S1). Neutral lipids accumulate in CLDs in enterocytes of the jejunum in the postprandial response to a dietary fat challenge, as defined by 2 h after an oral administration of 200 μl olive oil, in lean and DIO mice (Figure 1). The representative micrographs highlight the size disparity of CLDs that accumulate in enterocytes of lean (Figure 1A) compared to DIO mice (Figure 1B). CLDs in enterocytes of DIO mice are larger and have a different size distribution compared to lean mice in the postprandial response to a dietary fat challenge. The average CLD diameter was measured from 30 cells per mouse (*n* = 3 mice) from each diet group. No differences were observed in the number of CLDs per cell in lean and obese mice in the postprandial response to a dietary fat challenge (Figure 2A). DIO mice have significantly greater CLD diameter compared to lean mice in the postprandial response to a dietary fat challenge (Figure 2B). The average CLD size in DIO mice is 3.35 μm compared to the 1.88 μm average CLD diameter in lean mice. In support of this observation, CLDs in enterocytes from DIO mice have a statistically significant different size distribution compared to lean mice in the postprandial response to a dietary fat challenge, as determined by two-sample Kolmogorov–Smirnov test (Figure 2C).

**FIGURE 3:**
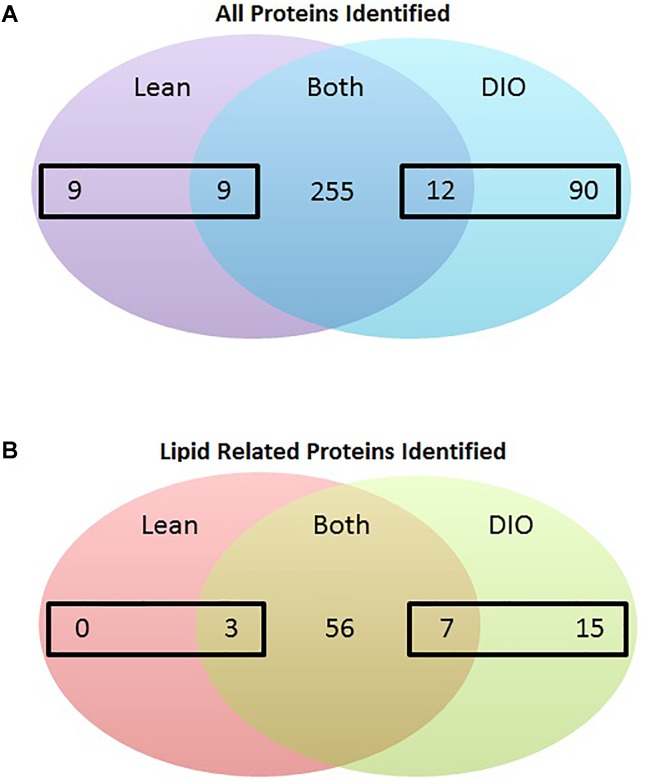
Number of proteins identified in the CLD fraction isolated from enterocytes of lean and DIO mice in the postprandial response to a dietary fat challenge. **(A)** 375 proteins were identified by tandem mass spectrometry analysis in the isolated CLD fraction from enterocytes of lean or DIO mice in at least three biological replicates. We identified 9 proteins present in the CLD fraction only from lean mice and 90 proteins present in the CLD fraction only from DIO mice after a dietary fat challenge. We identified 276 proteins in the CLD fraction of both lean and DIO samples. Of these 276 proteins, nine proteins had higher relative levels in the CLD fraction of lean samples, and 12 proteins had higher relative levels in the CLD fraction of DIO samples. **(B)** 81 identified proteins associated with lipid related GO terms. We identified 15 proteins associated with lipid related GO terms in the CLD fraction only from DIO mice. Of the 66 proteins associated with lipid-related GO terms in the CLD fraction of both lean and DIO mice, three proteins had higher relative levels in the CLD fraction of lean mice and seven proteins had higher relative levels in the CLD fraction of DIO mice. Differences in the relative levels of proteins identified in the CLD fraction of enterocytes between lean and DIO mice determined by *t*-test, *p* < 0.05 considered statistically significant.

To identify proteins that have potential to regulate CLD metabolism in lean and DIO mice in the postprandial response to a dietary fat challenge, we identified proteins in the CLD fraction isolated from enterocytes of mice from each diet group by LC-MS/MS. A protein was considered identified in a diet group if it was identified in at least three out of five biological replicates in at least one of the diet groups. We identified the relative levels of 375 proteins in the CLD proteomic profile (Supplementary Table S1). Of the 375 identified proteins, nine proteins were identified in lean only, 90 proteins identified in DIO only, and 276 proteins were found in both lean and DIO mice. Of the 276 proteins found common to lean and DIO mice, nine of them had higher levels in lean mice, and 12 of them had higher levels in DIO mice (Figure 3A).

The proteins identified in the CLD fraction of enterocytes from lean and DIO mice are associated with a variety of biological processes, defined by GO terms. The GO terms associated with the 375 identified proteins were compared to the complete mouse proteome and visualized using GOrilla. GOrilla highlights biological processes that are significantly enriched (*p* < 0.01 as determined by the GOrilla algorithm) within the CLD proteome dataset. Biological processes highlighted in red indicate the most significant enrichment. The proteins identified in the current analysis are associated with a wide variety of biological processes (Supplementary Figure S2). Biological process pathways that have the greatest enrichment include oxidation–reduction process (GO:0055114), cellular lipid catabolic process (GO:0044242), and organonitrogen compound metabolic process (GO:1901564). The complete annotation of biological processes enriched in the CLD proteomic data can be found in Supplementary Table S2.

To identify proteins that may contribute to CLD size and lipid accumulation, we limited our analysis to the 81 proteins identified that are associated with lipid related processes as determined using GOrilla (Supplementary Table S3). Proteins associated with lipid related processes are found at differential levels between the two diet groups and have been previously identified in other global or targeted intestinal CLD proteomic analyses (Lee et al., 2009; Bouchoux et al., 2011; Beilstein et al., 2013; Seyer et al., 2013; D’Aquila et al., 2015). Of the 81 lipid related proteins identified in the analysis, 25 are differentially present depending on diet group, and are either unique to lean or DIO mice or are present at statistically significant different levels in the CLD fraction of lean and DIO mice (Figure 3B and Table 1).

**Table 1:** Label free quantitation of proteins associated with lipid related GO terms.

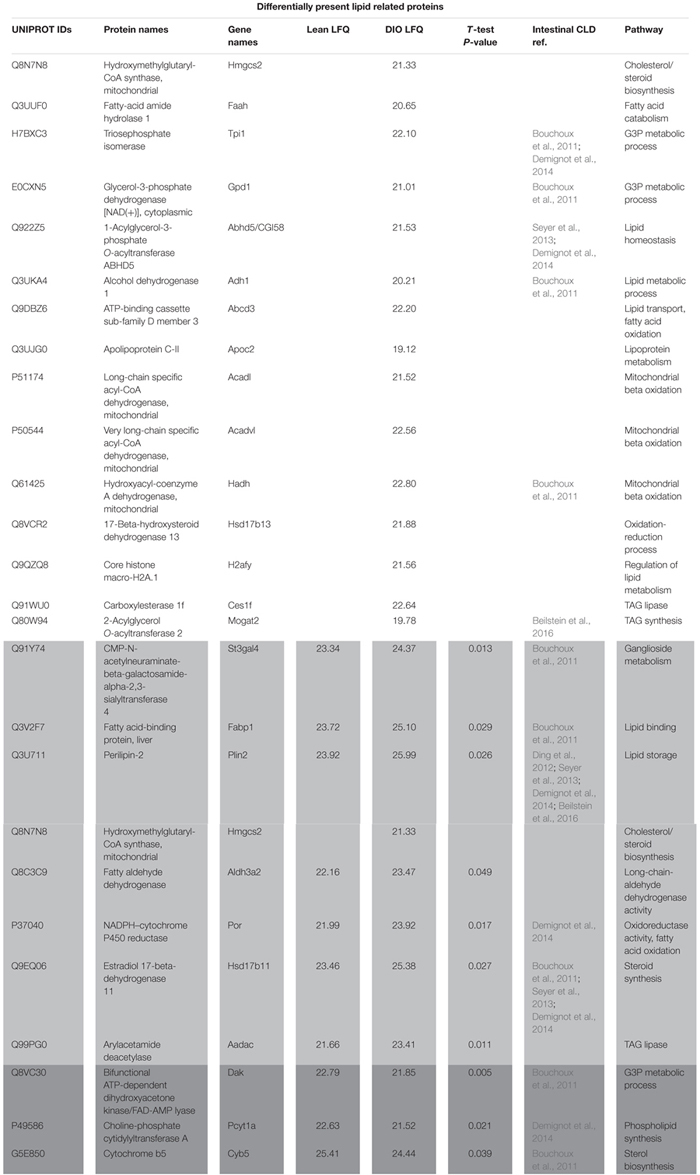

*Of the 375 identified proteins, 81 proteins are associated with lipid related GO terms as identified by GOrilla (Supplementary Table S3). Of the 81 lipid related proteins, 25 were found at differentially present levels in the CLD fraction from lean or DIO mice, as determined by t test. Proteins found exclusive to the CLD fraction of DIO mice are in white boxes, proteins found in both DIO and lean but with higher relative levels in DIO are in light gray, and proteins found in both DIO and lean but with higher relative levels in lean are in dark gray.*

**FIGURE 4:**
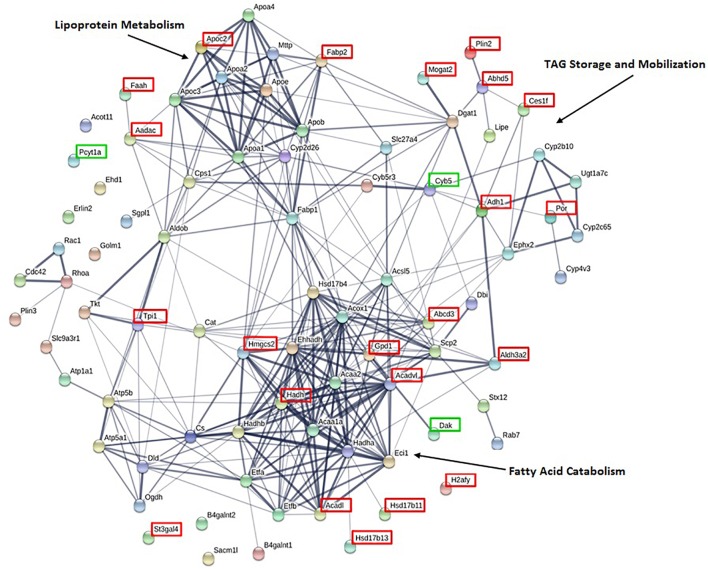
STRING analysis of proteins associated with lipid related GO terms highlight proteins clusters. Of the 375 identified proteins, 81 are associated with lipid related GO terms as identified by GOrilla. Proteins present at higher levels or exclusive to lean or DIO mice are indicated by a green or red box, respectively. Clusters of proteins with related functions are indicated.

To identify lipid related functional pathways enriched in the CLD proteome, we conducted a STRING analysis of the 81 lipid related proteins (Figure 4). The STRING analysis identified protein clusters related to lipoprotein metabolism, TAG storage and mobilization, and fatty acid catabolism. The 25 proteins that are found unique to or at higher levels in the CLD fraction of either lean or DIO samples are indicated by green and red boxes, respectively, and their relative levels are found in Table 1. Proteins found at higher levels in the CLD fraction of DIO samples are associated mainly with fatty acid catabolism, lipid synthesis, and lipid catabolism.

**FIGURE 5:**
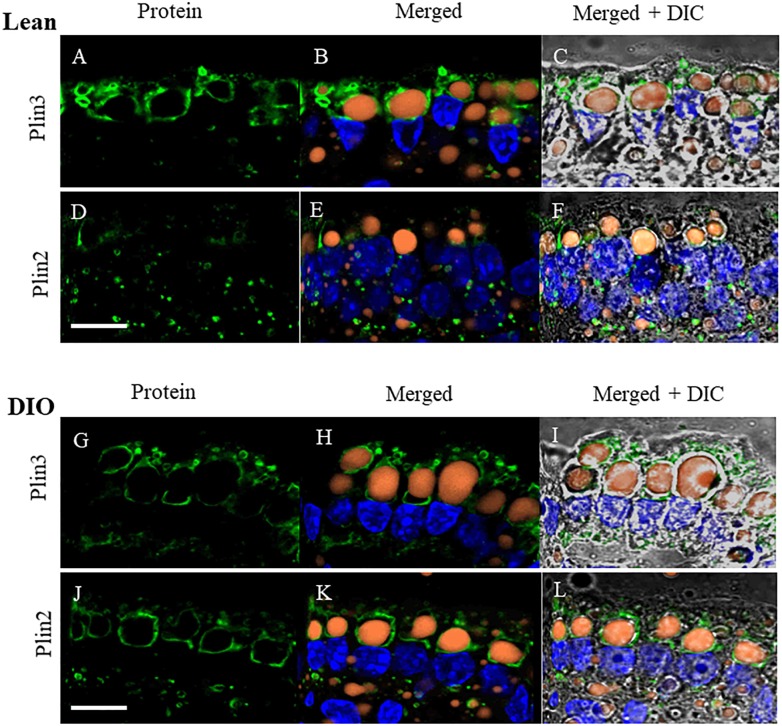
Plin3 and Plin2 localize to CLDs from lean and DIO mice in the postprandial response to a dietary fat challenge. **(A–C,G–I)** Plin3 (green) forms distinct ring like structures around BODIPY stained CLDs (orange) with a DAPI nuclear counterstain (blue) in a similar manner in lean and DIO mice. **(D–F,J–L)** Plin2 (green) forms more distinct ring like structures around CLDs in DIO **(J,K)** compared to lean mice **(D,E)** in the postprandial response to a dietary fat challenge. Scale bar is 10 μm.

**FIGURE 6:**
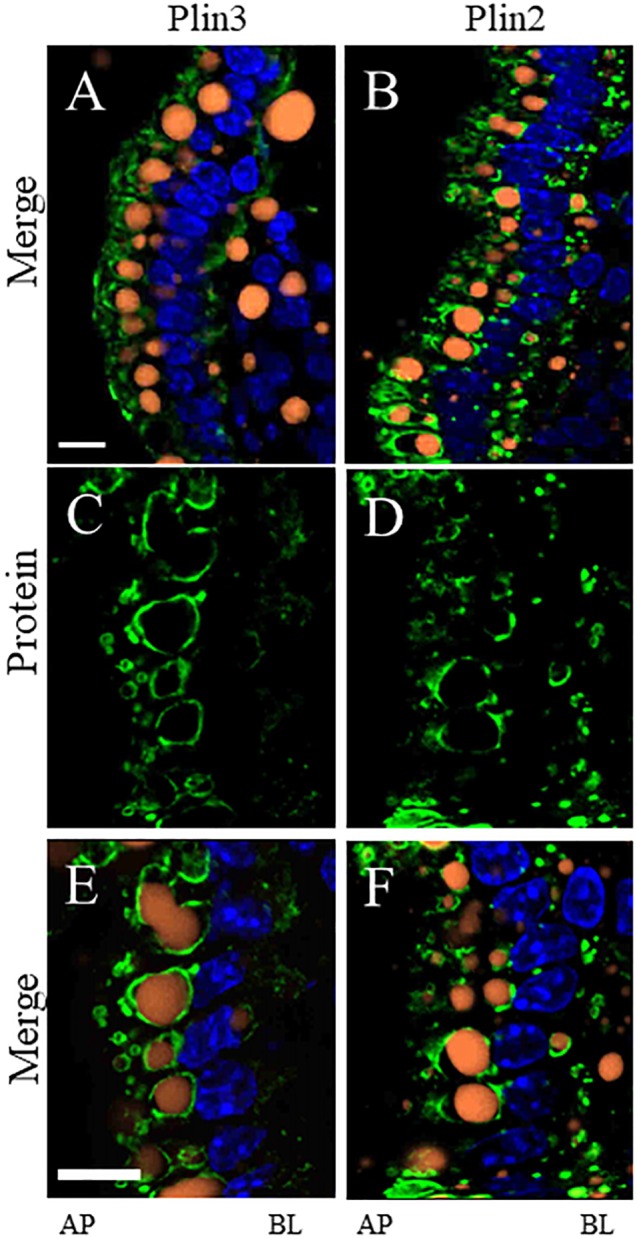
Plin3 localizes to CLDs on the apical (AP) side of the nuclei compared to Plin2 which localizes to CLDs on the AP and basolateral (BL) side of the nuclei in DIO mice in the postprandial response to a dietary fat challenge. Plin3 (green) localizes to CLDs (orange) on the AP side of the nuclei (blue) **(A,C,E)**. Plin2 (green) localizes to CLDs on the AP and BL side of the nuclei **(B,D,F)**. Panels **A,B** are low magnification views and panels **C–F** are higher magnification views. Scale bar is 10 μm.

To determine whether the proteins we identified by proteomic analysis in the CLD fraction of DIO and lean mice are in fact associated with the CLD, we used immunofluorescence confocal microscopy to visualize two proteins that were identified, Plin2 and Plin3 (Figure 5). Plin3 (Figure 5A–C,G–I) is found associated with CLDs from lean (Figure 5A–C) and DIO (Figure 5G–I) mice. Plin2 (Figure 5D–F,J–L) forms a more distinct ring-like structure around CLDs from DIO mice (Figure 5J–L) compared to lean mice (Figure 5D–F). These results validate that Plin2 and 3 associate with CLDs and that Plin2 has a different pattern of CLD association in lean and DIO mice. Interestingly, Plin2 and Plin3 localize to CLDs present in different regions of the cell in DIO mice (Figure 6). Plin3 (Figure 6A,C,E) localizes to CLDs primarily in the apical region (above the nucleus) of the cell and Plin2 (Figure 6B,D,F) localizes to CLDs both in the apical and basolateral side of the cell.

## Discussion

We investigated the effects of DIO on enterocyte CLD morphology and CLD proteome in the postprandial response to a dietary fat challenge. DIO mice have larger CLDs with a greater maximal size compared to lean mice (Figure 1, 2). Three hundred and seventy-five proteins representing a diverse range of functions were identified in the CLD fraction of enterocytes from lean and DIO mice in the postprandial response to a dietary fat challenge (Supplementary Tables S1, S2), and some of the 81 lipid related proteins are differentially present depending on diet group (Figure 3B and Table 1). Plin3 associates with CLDs from lean and DIO mice in a similar pattern while Plin2 forms more distinct ring-like structures around CLDs from DIO compared to lean mice in the postprandial response to a dietary fat challenge (Figure 5). Further, Plin2 and Plin3 are present on different pools of CLDs in DIO compared to lean mice in the postprandial response to a dietary fat challenge (Figure 6). These results demonstrate that DIO impacts CLD morphology and the CLD proteome.

The size of CLDs in enterocytes in the postprandial response to dietary fat is larger in DIO compared to lean mice. This may contribute to the observed decrease in TAG secretion rate in DIO compared to lean mice. Previous studies found that obese mice, including *ob/ob* and DIO mice, have decreased TAG secretion rates (Douglass et al., 2012; Uchida et al., 2012). However, analysis of intestinal mucosa by either biochemical analysis or coherent anti-scatter Ramen spectroscopy demonstrated no difference in TAG content between lean and DIO mice (Uchida et al., 2012). This analysis includes the quantification of TAG in all enterocyte pools. In this study, we observed TAG in a specific cellular pool, CLDs, by TEM and hypothesize that the larger CLD size is consistent with the observed TAG secretion rate, as CLDs serve as a storage depot for ultimate TAG secretion.

**FIGURE 7:**
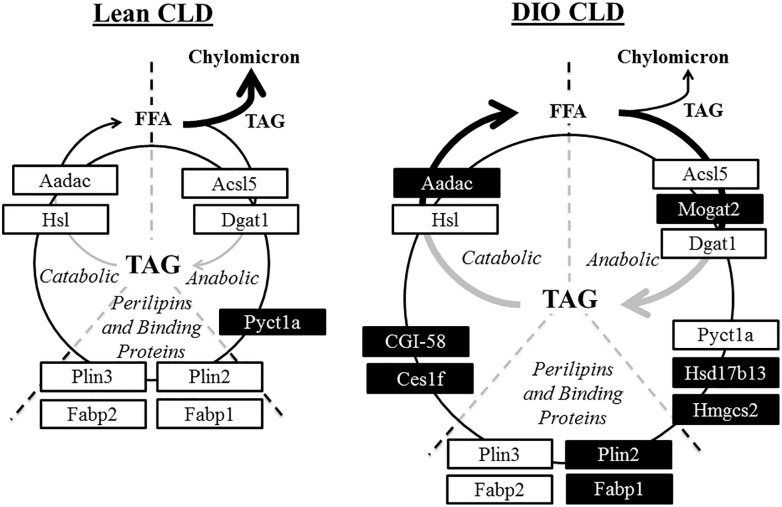
Hypothetical model of CLD metabolism in lean and DIO mice in the postprandial response to a dietary fat challenge. Proteins identified by proteomic analysis in the CLD fraction of enterocytes from lean and DIO mice in the postprandial response to a dietary fat challenge are associated with both anabolic and catabolic processes. Proteins at similar levels in the CLD fraction isolated from lean and DIO mice are in white boxes. Proteins either unique to or at higher levels in the CLD fraction of a diet group are in black boxes. This model supports the hypothesis of a cycle consisting of hydrolysis of stored TAG and concurrent TAG synthesis on CLDs in the postprandial response to a dietary fat challenge.

Proteins identified in the CLD fraction in this analysis agree with previous findings from other intestinal cell models (Table 1) and also expands upon knowledge of the CLD proteome. For example, choline-phosphate cytidylyltransferase A (Pcyt1a, or CCTα) was previously identified as associated with CLDs in other cell types (Krahmer et al., 2011). In addition, it was recently demonstrated that a lack of Pcyt1a in Caco2 cells increases TAG storage and CLD size and decreases TAG secretion after oleate treatment (Lee and Ridgway, 2018). In this study, Pcyt1a was present at lower relative levels in the CLD fraction of DIO compared to lean mice (Table 1). This may contribute to the larger CLD size and decreased TAG secretion rate observed in DIO mice in the postprandial response to a dietary fat challenge. We also identified proteins involved in complex lipid anabolism and catabolism in the CLD fraction of DIO and lean mice. For example, enzymes involved in TAG synthesis such as Mogat2, Dgat1, and Acsl5 were present in the CLD fraction, in addition to enzymes involved in TAG lipolysis such as Aadac, Hsl, Cgi-58, and Ces1f (Supplementary Table S1 and Table 1). Proteins involved in both anabolic and catabolic TAG pathways have been identified on CLDs in multiple cells types, including glycerol-3-phosphate acyltransferase 4 (GPAT4), 1-acylglycerol-3-phosphate *O*-acyltransferase 3 (AGPAT3), and DGAT2 involved in TAG synthesis in *Drosophila* S2 cells (Wilfling et al., 2013), and hormone sensitive lipase (Hsl), the carboxylesterase (Ces) family proteins, and arylacetamide deacetylase (Aadac) involved in TAG lipolysis in hepatocytes and adipocytes (Quiroga and Lehner, 2012; D’Andrea, 2016). Although the function of some of these proteins in CLD metabolism has been well characterized in other cell types, whether they are involved in CLD remodeling in enterocytes is unclear. In addition to enzymes involved in TAG turnover, we also identified proteins involved in fatty acid trafficking in the CLD fraction of both DIO and lean mice. Fatty acid binding protein (Fabp) 2 and Fabp1 are thought to traffic fatty acids to either anabolic or catabolic pathways (Gajda and Storch, 2015), for example, Fabp2 channels fatty acids toward TAG synthesis (Lagakos et al., 2011), while Fabp1 channels fatty acids to various metabolic pathways (Lagakos et al., 2011). Overall, the identification of proteins involved in TAG anabolism and catabolism in the CLD fraction provides a hypothetical model for the role of CLDs in directing the traffic of dietary lipid to either storage or secretion, potentially regulating the process of dietary fat absorption (Figure 7). Since localized lipid remodeling was not directly measured in this study, future investigation of the functional role these proteins play in TAG metabolism at the CLD is warranted.

Proteins identified in the CLD fraction of enterocytes from DIO mice in this analysis agree with previous findings for proteins identified in the CLD fraction of hepatocytes from mice fed a high fat diet that induces obesity and hepatic steatosis (Khan et al., 2015), suggesting a common response to high fat feeding. For example, apolipoprotein C2 (ApoC-II), fatty-acid amide hydrolase (Faah), ATP-binding cassette sub-family D member 3 (Abcd3), and very long-chain specific acyl-CoA dehydrogenase (Acadvl) are lipid related proteins found to be either unique to or at higher abundance in the CLD proteome of DIO mice compared to lean mice in both enterocytes (current study) and hepatocytes (Khan et al., 2015). These proteins are generally involved in catabolic lipid related processes, suggesting some common pathways in CLD metabolism may be similarly regulated in hepatocytes and enterocytes by high fat feeding. Future studies are required to determine whether and how they may influence CLD metabolism during high fat feeding.

Some CLD associated proteins localize to distinct CLD subpopulations. An unexpected observation made in this study was the localization of Plin2 and Plin3 to different CLD pools in enterocytes of DIO mice in the postprandial response to a dietary fat challenge (Figure 6). While this is the first observation of its kind in enterocytes, similar observations have been made in other cell types. For instance, members of the Plin family associate with adipocyte CLDs at different times during the maturation process (Wolins et al., 2005). Members of the cell death-inducing DNA fragmentation factor-α-like effector (CIDE) family of proteins localize to different CLD subpopulations in hepatocytes (Xu et al., 2016). Lastly, TAG synthesis enzymes localize to different CLD subpopulations in *Drosophila* S2 cells (Wilfling et al., 2013). A new model has recently been proposed that two groups of CLDs exist in the cell. These CLDs are described as initial lipid droplets and expanding lipid droplets each with a different CLD proteome that regulates CLD storage and growth (Kory et al., 2016). The current study provides supporting evidence for the presence of distinct CLD subpopulations with different configurations of proteins suggesting that each pool has a unique metabolism.

Lipid stored in certain pools of CLDs located in different regions of the enterocyte may be directed to specific metabolic fates, for example, fatty acid oxidation. Although fatty acid oxidation is a metabolic process mainly localized to mitochondria, proteins involved in fatty acid oxidation as well as other mitochondrial-associated functions are commonly identified in CLD proteomic studies (Goodman, 2008), as well as in this study (Table 1 and Supplementary Table S2). CLDs have been found to associate with mitochondria in multiple cell types (reviewed in Goodman, 2008; Aon et al., 2014; Schuldiner and Bohnert, 2017) and this association may provide fatty acids for oxidation and energy production or, recently, to use the ATP produced by mitochondria for TAG synthesis and CLD growth (Benador et al., 2018). In this study, we identified proteins involved in fatty acid oxidation in the CLD fraction of only DIO mice (Table 1). mRNA levels of genes involved in fatty acid oxidation (Kondo et al., 2006; de Wit et al., 2008; Douglass et al., 2012; Uchida et al., 2012) as well as fatty acid oxidation activity (Kondo et al., 2006) increases in enterocytes with chronic high fat feeding. In addition, we observed different pools of CLDs in DIO enterocytes in the postprandial response to a dietary fat challenge (Figure 6). Although fatty acid oxidation was not directly measured in this study, the identification of proteins involved in fatty acid oxidation in the CLD fraction of only DIO mice may reflect increased CLD–mitochondria interactions and an elevated level of fatty acid catabolism in DIO enterocytes in the postprandial response to a dietary fat challenge. Although it is unclear from this study which pool of CLDs may associate with mitochondria, these results provide evidence of potential CLD–mitochondrial interactions in enterocytes.

## Conclusion

In summary, we found that DIO alters CLD morphology and the CLD proteome in the postprandial response to dietary fat. CLDs from DIO mice are larger with a different size distribution compared to lean mice, which may contribute to their decreased TAG secretion rate. The CLD fraction from DIO and lean mice contains differentially present proteins, which may reflect differences in CLD metabolism in each diet group in the postprandial response to dietary fat. The proteins identified in the CLD fraction of DIO and lean mice are involved in both anabolic and catabolic lipid related processes, and provide a hypothetical model of TAG turnover in enterocytes. The pattern of Plin2 and Plin3 association with different pools of CLDs suggests that CLDs present in certain region of the enterocyte may have different metabolic fates. Overall, the results of this analysis provides a foundation for future work in studying CLD metabolism, and expands upon understanding the influence of DIO on dietary fat absorption.

## Author Contributions

KKB and TD conceived and designed the study. TD performed the experiments and wrote the first draft of the manuscript. KKB, TD, and ASZ analyzed the data, revised and wrote sections of the manuscript, and read and approved the final manuscript.

## Conflict of Interest Statement

The authors declare that the research was conducted in the absence of any commercial or financial relationships that could be construed as a potential conflict of interest.

## Supplementary Material

Figure S1 Body weight measurements of male C57BL/6 mice fed a high fat or low fat diet for 12 weeks.

Figure S2 GOrilla image of enriched GO terms associated with proteins identified by proteomic analysis. Biological processes highlighted in red indicate the most significant enrichment.

Table S1 Proteins identified in tandem mass spectrometry analysis of isolated CLDs from lean and DIO mice. Proteins are categorized by general function based on GO term classification.

Table S2 GOrilla analysis of enriched GO terms associated with proteins identified by proteomic analysis.

Table S3 GOrilla analysis of lipid related GO terms and associated proteins.

## Abbreviations

CLD: cytoplasmic lipid droplet
DGAT: diacylglycerol *O*-acyltransferase
DIO: diet induced obesity
GO: Gene Ontology
Plin: perilipin
TAG: triacylglycerol
