# Supplementary figures and images for: Diet Induced Obesity Alters Intestinal Cytoplasmic Lipid Droplet Morphology and Proteome in the Postprandial Response to Dietary Fat
Source: Front Physiol. 2019 Mar 5;10:180. doi: 10.3389/fphys.2019.00180 (PMC6413465; doi:10.3389/fphys.2019.00180)

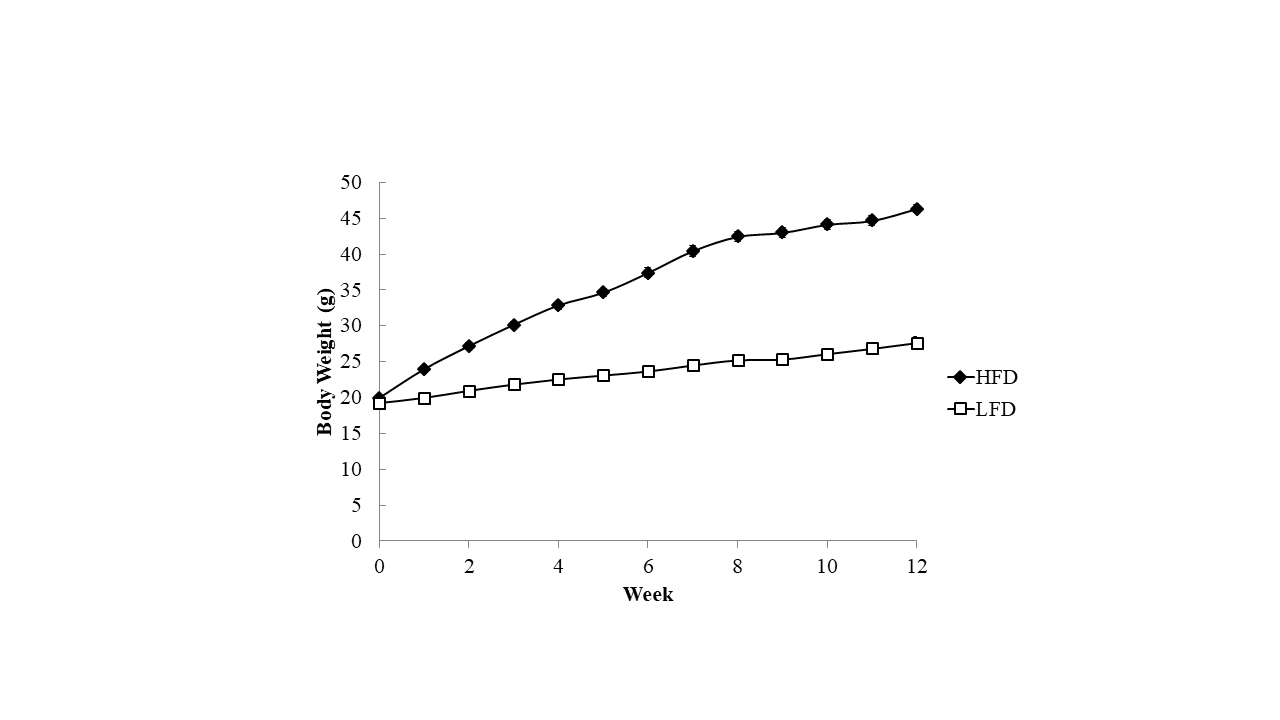

Supplement: Figure S1 — Body weight measurements of male C57BL/6 mice fed a high fat or low fat diet for 12 weeks. [file Image_1.TIF]

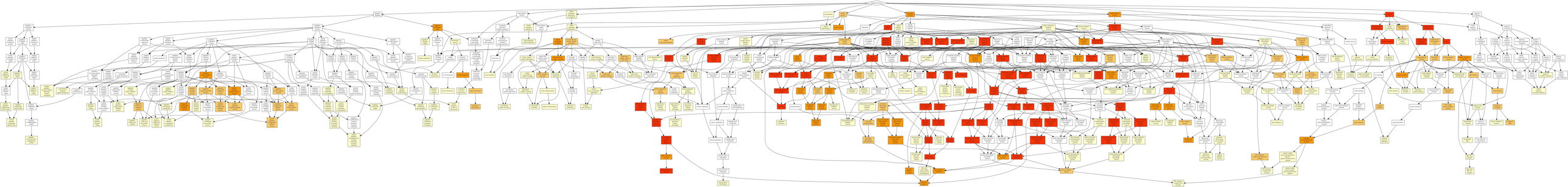

Supplement: Figure S2 — GOrilla image of enriched GO terms associated with proteins identified by proteomic analysis. Biological processes highlighted in red indicate the most significant enrichment. [file Image_2.JPEG]
